# Robust Control of PEP Formation Rate in the Carbon Fixation Pathway of C_4 _Plants by a Bi-functional Enzyme

**DOI:** 10.1186/1752-0509-5-171

**Published:** 2011-10-24

**Authors:** Yuval Hart, Avraham E Mayo, Ron Milo, Uri Alon

**Affiliations:** 1 Dept. of Molecular Cell Biology, Weizmann Institute of Science, Rehovot, Israel; 2 Dept. of Plant Sciences, Weizmann Institute of Science, Rehovot, Israel

## Abstract

**Background:**

C_4 _plants such as corn and sugarcane assimilate atmospheric CO_2_ into biomass by means of the C_4 _carbon fixation pathway. We asked how PEP formation rate, a key step in the carbon fixation pathway, might work at a precise rate, regulated by light, despite fluctuations in substrate and enzyme levels constituting and regulating this process.

**Results:**

We present a putative mechanism for robustness in C_4 _carbon fixation, involving a key enzyme in the pathway, pyruvate orthophosphate dikinase (PPDK), which is regulated by a bifunctional enzyme, Regulatory Protein (RP). The robust mechanism is based on avidity of the bifunctional enzyme RP to its multimeric substrate PPDK, and on a product-inhibition feedback loop that couples the system output to the activity of the bifunctional regulator. The model provides an explanation for several unusual biochemical characteristics of the system and predicts that the system's output, phosphoenolpyruvate (PEP) formation rate, is insensitive to fluctuations in enzyme levels (PPDK and RP), substrate levels (ATP and pyruvate) and the catalytic rate of PPDK, while remaining sensitive to the system's input (light levels).

**Conclusions:**

The presented PPDK mechanism is a new way to achieve robustness using product inhibition as a feedback loop on a bifunctional regulatory enzyme. This mechanism exhibits robustness to protein and metabolite levels as well as to catalytic rate changes. At the same time, the output of the system remains tuned to input levels.

## Background

A class of biological circuits was recently described with robust input-output relations [1-4]. In these systems, the output, such as the concentration or activity of a specific protein, is perfectly insensitive to variations in the concentrations of all of the system's components, and yet responsive to the system's input. Such robust input-output relations are difficult to achieve, because in most conceivable mechanisms the output is sensitive to variations in the concentrations of the circuit components.

At the heart of these robust mechanisms are bifunctional enzymes that catalyze two opposing reactions. The first example analyzed in detail appears in bacterial two-component signaling systems, in which a bifunctional receptor confers a robust input-output relationship by acting as both a kinase and a phosphatase of a response-regulator protein [1,2]. A second case of a bifunctional enzyme tied to robustness was studied in the glyoxylate bypass control of *E. coli *metabolism, in which the activity of isocitrate dehydrogenase is made robust by a bifunctional kinase/phosphatase [3]. A third example appears in the nitrogen assimilation system of *E. coli*, in which glutamine synthetase is controlled by a bifunctional enzyme that both adenylates and de-adenylates it [4].

Although all of these systems rely on bifunctional enzymes, each system does so with important differences. Thus, robustness relies in each case on the biochemical details of the system. In the two-component signaling case, robustness relies on an auto-kinase and phosphotransfer reaction by the receptor, as well as on the receptor phosphatase reaction being ATP-dependent. In the case of the glyoxylate bypass, robustness depends on saturating one of the sites of the bifunctional enzyme with substrate. And in the nitrogen assimilation case, robustness depends on avidity of the bifunctional enzyme to its multimeric substrate. Thus, in each case studied so far, there is a different detailed mechanism for robustness.

It is therefore of interest to describe additional systems with bifunctional enzymes, in order to discover new potential mechanisms for robustness. Here, we consider the plant carbon fixation cycle which employs a bifunctional kinase/phosphatase. The wealth of unusual biochemical features in this system makes it an interesting candidate for seeking a new mechanism for robustness. We next describe the reactions in this system and evidence for its robustness. We then propose a putative model for how robustness in this system can arise based on its biochemical architecture.

C_4 _plants such as corn and sugarcane use an enzymatic cycle to promote the assimilation of atmospheric CO_2_ into biomass. A key step in this cycle is the conversion of pyruvate to PEP by the enzyme pyruvate orthophosphate dikinase (PPDK) [5,6]. The activity of PPDK, namely the rate of PEP production, is controlled by light (because PPDK needs to be correlated with the photosynthesis rate, PPDK activity having the highest correlation with the photosynthetic rate, r = 0.96 [6]). Light level is encoded in the cell by the concentration of ADP: high ADP means low light, and low ADP means high light (see [7-11] and references therein). For simplicity, we will consider ADP levels as the input of the system, and PEP formation rate as its output.

The C_4 _pathway has three main types, two of which decarboxilate malate to pyruvate (by NADP-ME and NAD-ME enzymes) and then utilize PPDK as a crucial enzyme in the CO_2 _assimilation cycle [12]. Plants that employ these two types include maize, sugarcane, sorghum and millet. The third C_4 _type has the PPDK enzyme (and sequential cycle), but also has an additional decarboxylation pathway through PEPCK (phosphoenolpyruvate carboxykinase) which transforms oxaloacetate to PEP. In this third C_4 _pathway type, PPDK may be less crucial than in the first two types [12].

PPDK is one of the most abundant enzymes in the biosphere [13], constituting about 7-10% of the protein content of mesophyll cells [14]. It is maximally active as a homotetramer. When subjected to cold temperatures it dissociates into dimers and monomers, making it mostly inactive.

**Figure 1:**
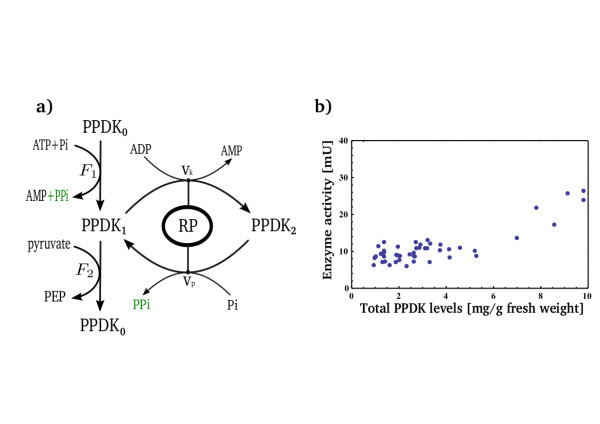
**The PPDK enzyme produces PEP and is regulated by the bifunctional enzyme RP**. Pyruvate, orthophosphate dikinase (PPDK_0_) uses ATP and Pi to produce phosphoenolpyruvate (PEP) from pyruvate. It does so in two stages: first, it auto-phosphorylate itself to its active form (PPDK_1_). Second, it transfers the phosphoryl group to pyruvate and returns to its natural form (PPDK_0_). Another regulatory cycle can phosphorylate the active form PPDK_1_ at a different residue to form PPDK_2_, the inactive form of PPDK. The second phosphorylation and de-phosphorylation are done by the bifunctional enzyme RP. This enzyme is regulated by ADP levels (an indication to photosynthetic rate). The products of the kinase/phosphatase activity of RP, AMP and PPi, are also the products of the auto-phosphorylation reaction of PPDK_0_ with ATP and Pi (b) Enzyme activity of PPDK is constant across a range of PPDK expression levels - as measured in a mutated strain of Maize (Zea Mays) by Ohta et al [18]. Mean levels of PPDK expression in non-transformants is measured to be 1.6 mg/g fresh weight). Enzyme activity changes only at extreme expression levels of PPDK (see text).

The reactions and regulation of PPDK have the following biochemical features (Figure 1). PPDK catalyzes the conversion of pyruvate to PEP in two steps: The first is auto-phosphorylation at a His residue (we denote this phosphorylated form PPDK_1_). The second step is a phospho-transfer reaction that transfers the phosphoryl to pyruvate to produce PEP. The autokinase reaction takes two phosphoryl groups from ATP and in the presence of Pi produces PPDK_1_, AMP and PPi. The two reactions are thus:

(1) $$\text{ATP}+\text{Pi}+\text{PPD}{\text{K}}_{0}\to \text{AMP}+\text{PPi}+\text{PPD}{\text{K}}_{\text{1}}$$

(2) $$\text{pyruvate}+\text{PPD}{\text{K}}_{\text{1}}\to \text{PEP}+\text{PPD}{\text{K}}_{0}$$

To regulate the activity of PPDK there exists a second phosphorylation/de-phosphorylation cycle. The auto-phosphorylated form of PPDK, PPDK_1_, can be phosphorylated a second time at a Thr residue. This doubly-phosphorylated form is an inactive form of the enzyme (denoted PPDK_2_). A bi-functional enzyme called Regulatory Protein (RP) catalyzes two opposing reactions: phosphorylation uses ADP as a substrate and its products are PPDK_2_ and AMP

(3) $$\text{PPD}{\text{K}}_{\text{1}}+\text{ADP}\to \text{PPD}{\text{K}}_{\text{2}}+\text{AMP}$$

whereas dephosphorylation uses inorganic phosphate Pi to produce PPi:

(4) $$\text{PPD}{\text{K}}_{\text{2}}+\text{Pi}\to \text{PPD}{\text{K}}_{\text{1}}+\text{PPi}$$

The two phosphorylation steps of PPDK are sequential, meaning that the second phosphorylation of PPDK at the Thr residue can happen only on the auto-phosphorylated form PPDK1, i.e. only after PPDK is in its active form and ready to phosphorylate pyruvate to produce PEP [15,16]. The bifunctional enzyme RP is inhibited in a competitive manner by PPi [17].

A recent experimental study indicates that the activity of PPDK1 (the PEP formation rate) is insensitive to variations in PPDK protein levels [18]. Ohta et al. transformed a cold-tolerant PPDK gene into maize. The transformation yielded 48 strains each with a different expression level of PPDK. The strains were then measured for PPDK enzyme activity. These measurements show that enzyme activity is nearly insensitive to increasing or decreasing PPDK expression levels: there was only about a 20% change of PEP formation rate despite a 5.7 fold variation in PPDK levels [18] (for examples in other metabolic enzymes see [19]). This suggests that PPDK activity is regulated in a way to ensure a robust PEP formation rate (see Figure 1b). The nature of this mechanism remains unknown.

In this study, we demonstrate that the detailed features of the system can work together to provide input-output robustness. We propose a mechanism that makes the output (rate of PEP formation) robust to wide variations in the concentration of all of the system components, including protein levels (RP and PPDK) and substrate metabolite levels (ATP, pyruvate). Despite this robustness, the rate of PEP formation is still sensitive to its input signal ADP which corresponds to photosynthetic activity (light/dark). The mechanism proposed in this study is based on avidity of the bifunctional enzyme RP to PPDK tetramers. It also depends on a product-inhibition feedback effect of a PPDK product (PPi) on RP's catalytic rates. We also detail the conditions in which robustness breaks down, such as extreme values of the input ADP, or ultra-low levels of substrates or proteins.

## Results

### A mechanism based on bifunctional enzyme avidity and product inhibition suggests robustness of PEP formation rate

We present a mechanism for robustness in the system based on its known biochemical features. The outline is as follows: we first note that the tetramer structure of PPDK makes possible an avidity effect, in which RP primarily acts when it is bound at the same time to two different monomers on the same tetramer. We then show that this avidity effect allows the system to reach steady-state only if the specific rates of the kinase and phosphatase reactions of RP are exactly equal. Finally, we note that such tuning of specific rates is made possible by a feedback loop, in which the rate of PEP formation affects RP rates by product-inhibition (through the shared metabolite pyrophosphate). The upshot is that the PEP formation rate (the output of the system) depends only on the input signal (ADP, which corresponds to light level), and not on any of the protein levels (PPDK, RP), levels of metabolite substrates (pyruvate, ATP) or on PPDK catalytic rate. The full set of equations of the mechanism is shown in additional file 1. The following description aims to allow an intuitive understanding of the mechanism.

#### The avidity effect in RP action

We denote the non-phosphorylated form of PPDK by PPDK_0_, the phosphorylated form at the His residue by PPDK1 and the doubly phosphorylated form at the His and Thr residues by PPDK_2_. Only PPDK_1_ is active and catalyzes the production of PEP.

The bifunctional enzyme RP has two domains, one for kinase and the other for phosphatase activity [20,21]. This two-domain structure, together with the tetrameric form of its substrate PPDK, provides for a cooperative binding effect known as the *avidity *effect. Avidity results when one domain of RP, the kinase domain, binds a PPDK_1_ subunit and the other domain binds a PPDK_2_ subunit on the same tetramer.

We name the situation where RP simultaneously binds two PPDK subunits as the *ternary complex*, [PPDK_1_ RP PPDK_2_]. The situation where RP binds only one domain is termed a binary complex. The binary complexes are [RP PPDK_1_] and [RP PPDK_2_]. Thus, at steady-state, the total RP kinase activity equals the RP phosphatase activity, and includes the contribution of both binary and ternary complexes

(5) $$\begin{array}{c} {\text{V}}_{\text{k}}\left(\text{ADP}\right)\;\left(\left[\text{RP PPD}{\text{K}}_{\text{1}}]+[\text{PPD}{\text{K}}_{\text{1}}\;\text{RP}\;\text{PPD}{\text{K}}_{\text{2}}\right]\right)=\\ \;={\text{V}}_{\text{p}}\text{(ADP)}\left(\left[\text{RP}\;\text{PPD}{\text{K}}_{\text{2}}]+[\text{PPD}{\text{K}}_{\text{1}}\text{RP}\;\text{PPD}{\text{K}}_{\text{2}}\right]\right) \end{array}$$

where V_k_(ADP) and V_p_(ADP) are the specific catalytic activities of the two domains of RP. These rates depend on the input ADP [17].

Due to the avidity effect, however, the ternary complex is highly favored relative to binary complexes. Once RP binds one subunit of PPDK, for example PPDK_1_, the effective local concentration of a neighboring subunit (PPDK_2_) is increased. As a result, the on-rate for the second binding is very high (typical avidity effects show an on rate that is 100 times or more larger than the first binding rate [4,22]). Unbinding is rare, because both subunits need to unbind at the same time for RP to leave the tetramer.

**Figure 2:**
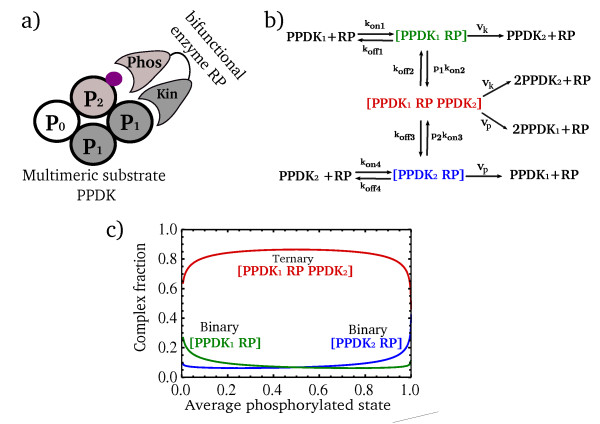
**Avidity favors a ternary complex in which the two domains of RP bind to two adjacent monomers in the PPDK multimer**. (a) Illustration of the two domains of an RP enzyme binding adjacent PPDK monomers in a PPDK tetramer. The phosphoryl-addition domain binds PPDK_1_, whereas the phosphoryl-removal domain binds PPDK_2_. This double-binding results in high avidity. (b) Reactions for the formation of binary and ternary complexes of RP and PPDK. When one domain of RP binds a monomer, the rate for binding an adjacent monomer by the second domain, kon2 and kon3, is very large. (c) The fraction of RP found in a ternary complex with two adjacent monomers [PPDK_1_ RP PPDK_2_] is higher than the fraction found in binary complexes with only one monomer [PPDK_1_ RP] or [PPDK_2_ RP], over most of the range of PPDK phosphorylation levels. In this plot the parameters were normalized to koff1 and total RP levels such that kon1 = kon4 = 0.01[koff1/RP], koff4 = koff1, kon2 = kon3 = 200[koff1], koff2 = koff3 = 1[koff1], V_k _= V_p _= 0.01[koff1] and total PPDK/total RP = 100 [11,13,33]. The complex fractions were calculated with a model that takes into account the spatial configurations of PPDK subunits (see Methods).

Avidity therefore ensures that, as long as both PPDK_1_ and PPDK_2_ forms are present on the same tetramer, the ternary complex is the prevalent complex in the system (see Figure 2). As a result, both phosphorylation and de-phosphorylation catalyzed by RP occur mainly in the ternary complex. This applies also in a more detailed model, presented in the last section of the results, that takes into account the spatial organization of the three possible states of PPDK subunits along the tetramer. Thus, we assume that, as a first approximation, we can neglect the binary complexes in Eq.(5), to find that the condition for steady-state is equality between the rates catalyzed by ternary complexes:

(6) $${\text{V}}_{\text{k}}\left(\text{ADP}\right)\left[\text{PPD}{\text{K}}_{\text{1}}\;\text{RP}\;\text{PPD}{\text{K}}_{\text{2}}\right]={\text{V}}_{\text{p}}\left(\text{ADP}\right)\left[\text{PPD}{\text{K}}_{\text{1}}\;\text{RP}\;\text{PPD}{\text{K}}_{\text{2}}\right]$$

When the ternary complex level is non-zero, one can cancel it out from both sides of the equation. This means that steady-state requires equal specific kinase and phosphatase rates for the bifunctional enzyme RP:

(7) $${\text{V}}_{\text{k}}\left(\text{ADP}\right)={\text{V}}_{\text{p}}\left(\text{ADP}\right)$$

This is a requirement that cannot generally be met, because the input signal ADP changes V_k _and V_p _in opposite directions (except for a single value of ADP, V_k _and V_p _are generally unequal). Thus, steady state requires an additional layer of regulation. We next describe an effect due to product inhibition, which can satisfy the steady-state condition, and turns out to provide robustness.

#### Tuning of RP velocities can be achieved through a product-inhibition feedback loop

Note that the products of the auto-kinase reaction of PPDK, AMP and PPi, are also the products of the RP reactions: AMP is the product of the kinase reaction, and PPi the product of the phosphatase reaction. In the present view, these features can help to form a robust mechanism, because they provide a feedback loop between PPDK and RP activities. This feedback is due to the phenomenon of *product inhibition *[19] of RP. The phosphatase activity of RP has been found to be inhibited by its product PPi, following a Michaelis-Menten like inhibition curve [17]

(8) $${\text{V}}_{\text{p}}\left(\text{ADP},\text{PPi}\right)={\text{V}}_{\text{p}0}\left(\text{ADP}\right)∕\left(\text{1}+\left[\text{PPi}\right]∕{\text{K}}_{\text{i},\text{PPi}}\right)$$

Where K_i_,_p__p__i_ = 160 μM is the inhibition constant [17] and V_p__0_(ADP) is the maximal phosphatase velocity. Thus, the more PPi in the cell, the lower is the phosphatase activity of RP. Experiments suggest that the kinase reaction of RP is not measurably inhibited by the second product AMP (K_i_,_A__M__P_ > 2 mM, [17]).

Since PPi is produced by PPDK, and inhibits RP, it can link these two enzyme activities. For this to happen, however, the concentration of PPi in these cells must be determined mainly by PPDK, and not by the hundred or so other reactions that produce PPi [23]. The situation in these plant cells might be special, however, because of the huge amount of PPDK enzyme (7-10% of total protein). We therefore assume that the main production source of PPi is the PPDK auto-kinase activity, and neglect to a first approximation all other PPi sources (see also additional file 2). The concentration of PPi in such a case is given by the balance of its production rate by the PPDK_0_ auto-kinase reaction, F_1_(ATP,Pi,PPi), and its degradation at rate α

(9) $$\text{d}\left[\text{PPi}\right]∕\text{dt}={\text{F}}_{\text{1}}-\alpha \left[\text{PPi}\right]$$

Solving this results in a steady-state concentration of PPi that is proportional to the production rate from the auto-kinase reaction (F_1_), [PPi] = F_1_/α. This is important because at steady-state each auto-kinase reaction corresponds to one PEP formation reaction: the phosphate is transferred from PPDK onto pyruvate to produce PEP. Because of this stoichiometric relationship, the system output, PEP formation rate F_2_, is equal to the production rate of PPi from the auto-kinase: F_2_= F_1_. These considerations link the PEP formation rate, F_2_, to the PPi concentration,

(10) $${\text{F}}_{\text{2}}=\alpha \left[\text{PPi}\right]$$

Using this relation in Eq.(8), we see that product inhibition of RP by [PPi] leads to the following connection between the systems output F_2 _and the RP phosphate rate:

(11) $${\text{V}}_{\text{p}}\left(\text{ADP}\right)={\text{V}}_{\text{p}0}\left(\text{ADP}\right)∕\left(\text{1}+{\text{F}}_{\text{2}}∕{\text{F}}_{0}\right)$$

**Figure 3:**
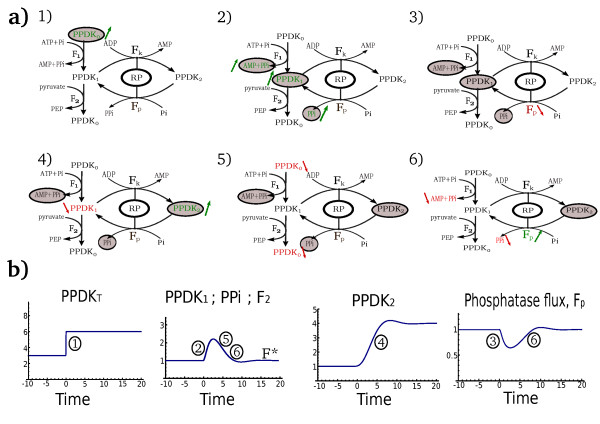
**Combination of avidity with a product-inhibition negative feedback loop provides robustness to the PEP formation rate**. **a) **A schematic illustration of how the PPi negative feedback leads the output PEP formation rate back to its robust level upon a step addition of PPDK protein. (1) An increase in total PPDK levels leads to a rise in PPDK_0_ levels. In turn, (2) PPDK_1 _and PPi rise. (3) PPi rise leads to inhibition of the phosphatase activity of RP (product inhibition) and (4) to a rise in PPDK_2_ levels, lowering PPDK_1_ amounts. (5) The decrease in PPDK_1_ yields a decrease in PPDK_0_ and thus leads to (6) a decrease in PPi and rise of RP phosphatase rate. The robust formation rate, F*, is again attained. Since the avidity mechanism places both the kinase and phosphatase reactions from the same complex (ternary complex), steady-state requires only that their rates are equal (regardless of protein levels). Thus, steady state formation rate goes back to its original state, while the extra amount of PPDK is routed to the PPDK_2_ state, which acts as a buffer. Grey circles indicate deviations from the initial steady-state and green (red) arrows indicate increased (decreased) levels. **b) **Schematic illustration of the system's dynamics upon an increase in PPDK total amounts. The numbers in the different plots mark the phases of the system's adaptation back to the robust solution as marked in **a)**.

Where F_0 _= α Ki,PPi. This closes a negative feedback loop: the higher the PEP formation rate F_2_, the lower the phosphatase activity of RP, and thus the more PPDK in its inactive form PPDK_2_, leading to lower PEP formation rate (see Figure 3). This loop leads the PEP formation rate to a point at which the RP kinase and phosphatase activities are equal (Eq.(7)). Using Eq.(11), we find that this steady state PEP formation rate is

(12) $${\text{F}}_{\text{2},\text{st}.\text{st}}={\text{F}}^{*}=\alpha \;{\text{K}}_{\text{i},\text{PPi}}\left({\text{V}}_{\text{p}0}\left(\text{ADP}\right)∕{\text{V}}_{\text{k}}\left(\text{ADP}\right)-\text{1}\right)$$

This is the main result of the present analysis. The output formation rate F* *does not depend on the concentrations of the proteins in the system, RP and PPDK. It also does not depend on any of the substrate metabolites, ATP, pyruvate, PEP and AMP*. The formation rate is thus robust to these potentially fluctuating concentrations as been also suggested by studies in leaves and isolated chloroplasts showing no clear relation between PPDK activity and changes in ATP, AMP, pyruvate and PEP levels (reviewed in [10] and references therein, see also additional file 2). Despite this robustness, the output rate is controlled by the input signal ADP, which corresponds to light levels.

The magnitude of the output (PEP formation rate) in this mechanism is given by the product of the PPi product-inhibition constant and the PPi degradation rate, F_0 _= α Ki,PPi. We note that pyrophosphatases are abundant in the chloroplast [24], providing a fast hydrolysis specific activity of 40 μmol/mg chl/min [25], yielding α ≈100 [1/sec]. Since Ki,PPi = 160 μM [17] one finds a rate of about F_0 _= 10^8 ^reactions/second per chloroplast (for chloroplast of size 20 μm^3 ^[26]). This rate magnitude makes sense: the C_4 _cycle in these plant cells assimilates about 10^7^-10^8 ^carbon atoms in the form of CO_2_ per second per chloroplast at daylight [27,28] (see additional file 2 for more details).

### Limits of robustness

We also studied the conditions in which robustness might break down. The model suggests three cases: The first potential condition for loss of robustness is when there is not enough total PPDK enzyme or substrates to provide the robust rate F* of Eq.(12). The second includes conditions of very low or very high input signal, in which the binary complexes in Eq.(5) cannot be neglected, and avidity is no longer a dominant effect. The third condition for loss of robustness occurs when total PPDK levels are extremely high such that its activity cannot be regulated due to shortage in the phosphorylation substrate (ADP levels). We now briefly analyze these conditions.

**Figure 4:**
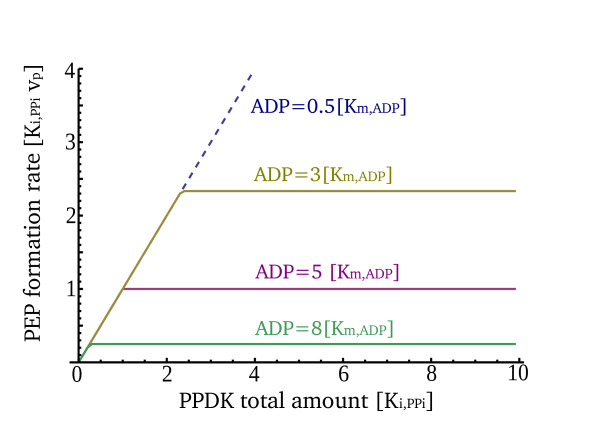
**The PEP formation rate is robust to PPDK levels and sensitive to ADP levels**. Numerical simulations of the PPDK system show that the robust solution has a range of validity. At very low ADP levels (blue dashed line), the photosynthetic rate is high and the system is fully active, all PPDK subunits are in their PPDK_1_ form. Then, PEP formation rate is dependent on PPDK total amounts. At medium levels of ADP (gold, purple and green), PEP formation rate is robust to PPDK total amounts and depends solely on ADP levels (the signal). This rate is dependent on PPDK total amount only when PPDK levels are too low to provide the robust solution, F* (see text). Finally, at high ADP levels (darkness), the system shuts down and almost all PPDK subunits are inactivated (PPDK_2_), which yields a very low basal formation rate level. Loss of robustness also occurs when PPDK levels are extremely high such that ADP levels are not sufficient to phosphorylate the added active PPDK units (see text and Figure 1b)

The first type of conditions in which robustness does not occur is when there is not enough total PPDK enzyme or substrates (ATP, pyruvate) to provide the robust PEP formation rate F* given by Eq.(12). For example, if substrate or PPDK levels are zero, one must have F_2 _= 0. Solution of the model shows that when one of these factors (total PPDK, pyruvate or ATP levels) goes below a threshold concentration (equal to its minimal concentration needed to reach F*), all of PPDK becomes active (PPDK_2_ = 0). The formation rate F_2 _is then linear in PPDK1, F_2 _= V1(pyr) PPDK1. In this state, the rate depends on protein and metabolite levels and robustness is lost. As soon as PPDK and/or substrate levels become high enough to reach F*, robustness is restored (see Figure 4).

The second case for loss of robustness is extreme input levels in which the binary complexes are not negligible compared to ternary complexes. Avidity requires that PPDK exist on the same tetramer in both PPDK1 and PPDK_2_ forms. However, in extreme high or low signal (ADP) levels, this does not apply. In these conditions, one can no longer neglect the effects of binary complexes (see Methods and additional file 2). At very low ADP levels (very high light), most PPDK is active and PPDK_2_ monomers are rare. Ternary complexes are scarce because they require PPDK_2_.

We estimate that robustness begins to erode at light levels below 50 μE m^-2 ^s^-1 ^or above 800 μE m^-2 ^s^-1^, which is also the mean photosynthetic photon flux at daylight [28,29]. Thus robustness is found between an upper and lower bounds on the light input (and its corresponding ADP encoding), as illustrated in Figures 2 and 4.

Robustness also breaks down at an extreme case when total PPDK levels exceed ADP concentration (PPDK_*T *_>> ADP), a condition that physiologically cannot be met due to the very high levels of this protein. In this case, cellular ADP levels are too low to allow further phosphorylation of the excess PPDK_1_. Consequently, the rate of PEP formation will be linearly dependent on PPDK total amounts (see Figure 1b, high end of the x axis and additional file 2).

We also note that to be feasible, the robust mechanism must admit a positive and stable solution. Exact solution of the model shows that this corresponds to the condition V_k_ < V_p_0, namely that the RP kinase rate is smaller than the phosphatase maximal rate (the rate in the absence of inhibition).

### A model for the spatial arrangement of PPDK subunits based on avidity predicts a bimodal distribution of phosphorylated and unphosphorylated tetramers

Finally we analyze the detailed configurations of PPDK states within PPDK tetramers, when the robust mechanism is active. The robust mechanism involves the RP cycle catalyzed primarily by RP bound to two adjacent subunits of PPDK, one in PPDK_2_ form and the other in PPDK_2_ form. The abundance of this ternary complex relative to binary complexes is due to the avidity effect.

When RP carries out a reaction, it changes the state of one of the two subunits that it binds: changing PPDK_1_ to PPDK_2_ or vice verse. It thus converts adjacent PPDK_1_- PPDK_2_ subunits either to two adjacent PPDK_1_ subunits, or two adjacent PPDK_2_ subunits.

**Figure 5:**
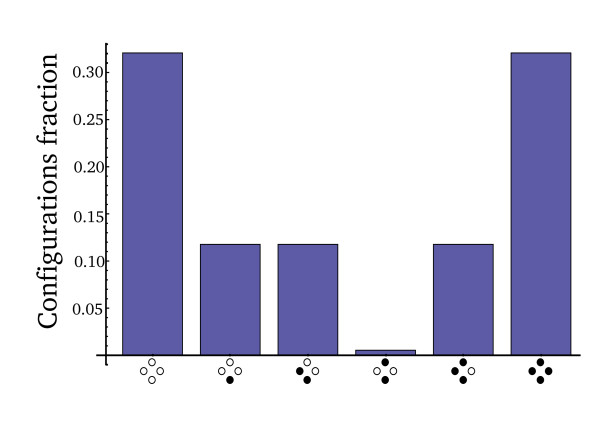
**Avidity model predicts a bimodal distribution of PPDK subunit phosphorylation states**. Subunit state configurations were evaluated using a model of spatial arrangements of PPDK subunits. Presented is the steady-state fraction (see Methods) of each configuration of the PPDK tetramer. We focused only on configurations of either PPDK_1_ (marked in white) or PPDK_2_ (marked in black) subunits. Similar results hold for configurations where one or more subunits are in the PPDK_0_ state (see additional file 2). Model parameters are: V_p _= V_k _= 0.2 [koff1], kon2 = 200 [koff2] and total PPDK/total RP = 100 [11,13,33].

The action of RP therefore tends to convert neighboring subunits that have different forms to the same form. Analyzing this in a detailed model that tracks the different configurations of tetramers (see Methods), we find that the dynamics reaches a steady-state in which the configuration distribution resembles a bimodal distribution. In this distribution, tetramers tend to be made of all PPDK_1_ or all PPDK_2_ subunits (Figure 5). These forms are slowly converted to other forms by RP binding to a single monomer (binary complex). The rarest forms are those with adjacent PPDK_1_- PPDK_2_ states, arranged in a "checkerboard" pattern. A quantitative analysis of the configuration probability distribution and its effect on the ratio of ternary to binary reactions is presented at the Methods section.

We also studied the effect of a three-state model on the different configurations, with 3 possible states for PPDK subunits, namely PPDK_0_, PPDK_1_ and PPDK_2_. We find that for the system to attain robustness it is beneficial that the two steps of the phospho-transfer have different rates. Only if the auto-phosphorylation of PPDK is faster than the phospho-transfer to pyruvate, the majority of the PPDK pool will transition between the PPDK_1_ and PPDK_2_ states and the ternary complex will dominate the modification reactions. Otherwise, the majority of the configurations will be in the PPDK_0_ state which hampers the probability for a ternary complex to exist. In-vitro measurements suggest that the auto-phosphorylation reaction is 1.5 faster than the phospho-transfer reaction [30]. We find that this is sufficient for the avidity reactions to dominate the process, and for robustness to result.

## Discussion

We presented a putative mechanism for robustness in the PPDK system of the C_4 _pathway in plants. The mechanism depends on avidity of the bifunctional enzyme RP to its multimeric substrate PPDK, and on a product-inhibition feedback loop that couples the system output PEP formation rate to the activity of the bifunctional regulator. The resulting output, PEP formation rate, is made insensitive to variations in substrates and protein levels. Despite this robustness, the output formation rate can be tuned by the input of the system, light levels encoded by ADP concentration.

Robust control of PEP formation rate in the C_4 _cycle might be important in order to synchronize its action with the photosynthesis rate. The C_4 _pathway allows plants to increase their internal CO_2_ concentration near the carbon fixing apparatus. Good regulation can ensure an optimal balance of resources in the plant and help avoid reactive oxygen species accumulation [31].

Robustness is predicted to break down at very high or very low light levels, or when PPDK concentration or the concentrations of its substrates are too low to provide the robust solution. In these cases the output formation rate becomes proportional to PPDK levels and catalytic activity. Darkness leads to a shutdown of PEP production (perhaps to a low basal level) to match the lack of photosynthesis. Shortage in substrates or PPDK enzyme leads to maximal activity of PPDK [17]).

Our model also predicts that the robust rate solution F* (Eq.12) does not depend on the catalytic rate of PPDK. Thus, the PEP formation rate can be insensitive to temperature effects on PPDK specific activity. This feature of the model may explain the robust activity of PPDK observed in Maize across temperatures from 28°C to 45°C [32].

One interesting question raised by the present finding is why should each cell be robust, when there are so many cells in the plant tissue that errors might be averaged away? A robust PEP formation rate in each mesophyll cell, despite the fluctuations averaging ability of the entire tissue, suggests that each cell may require the optimal rate level at each given conditions. Being above or below this optimal level may cause damage to the cell, or reduce its growth ability. For example, ATP is needed both for carbon fixation and for biosynthesis. An unnecessary consumption of ATP by an error of too much C_4 _cycle carbon fixation rate could hamper biosynthesis. Similarly, reduced PEP formation rate (and thus higher levels of ATP) would reduce carbon fixation rate and therefore cell growth. Because of such effects, fluctuations at the single cell level may not average out but rather decrease the fitness of the entire tissue.

The PPDK mechanism is readily testable by experiments that test PEP formation rate as a function of enzyme and substrate levels in plants. Experiments can also test the breakdown of robustness predicted at extreme light levels and very low levels of enzyme or substrates. One can also test the importance of avidity by studying mutant mono-functional versions of the bifunctional enzyme RP [4]. More generally, the suggested mechanism provides a context for the unusual set of biochemical features in this system. When considered together, these features have the potential to perform a systems level function: providing robustness with respect to fluctuating components and at the same time responsiveness to the input signal.

## Conclusions

The presented PPDK mechanism is a new way to achieve robustness using product inhibition as a feedback loop on a bifunctional regulatory enzyme. This mechanism exhibits robustness, being insensitive to variations in protein and metabolite levels as well as to catalytic rate changes. At the same time, the output of the system remains tuned to input levels.

## Methods

### Mathematical model of the ternary complex avidity

We used mass-action kinetics to describe the model illustrated in Figure 2:

(13) $$\begin{array}{c} \text{d}\left[\text{PPD}{\text{K}}_{\text{1}}\;\text{RP}\right]∕\text{dt}=\text{kon1}\;\text{PPD}{\text{K}}_{\text{1}}\;\text{RP}-\left(\text{koff1}+{\text{V}}_{\text{k}}+\text{kon2 p1}\right)\;\left[\text{PPD}{\text{K}}_{\text{1}}\;\text{RP}\right]\\ +\text{koff2}\left[\text{PPD}{\text{K}}_{\text{1}}\;\text{RP}\;\text{PPD}{\text{K}}_{\text{2}}\right]=0 \end{array}$$

(14) $$\begin{array}{c} \text{d}\left[\text{PPD}{\text{K}}_{\text{2}}\;\text{RP}\right]∕\text{dt}=\text{kon4 PPD}{\text{K}}_{\text{2}}\;\text{RP}-\left(\text{koff4}+{\text{V}}_{\text{p}}+\text{kon3 p2}\right)\;\left[\text{PPD}{\text{K}}_{\text{2}}\;\text{RP}\right]\\ +\text{koff3}\left[\text{PPD}{\text{K}}_{\text{1}}\;\text{RP}\;\text{PPD}{\text{K}}_{\text{2}}\right]=0 \end{array}$$

(15) $$\begin{array}{c} \text{d}\left[\text{PPD}{\text{K}}_{\text{1}}\;\text{RP}\;\text{PPD}{\text{K}}_{\text{2}}]∕\text{dt}=\text{kon2}\;\text{p1}\;[\text{PPD}{\text{K}}_{\text{1}}\;\text{RP}]+\text{kon3 p2}\;[\text{PPD}{\text{K}}_{\text{2}}\;\text{RP}\right]-\\ \left(\text{koff2}+\text{koff3}+{\text{V}}_{\text{k}}+{\text{V}}_{\text{p}}\right)\left[\text{PPD}{\text{K}}_{\text{1}}\;\text{RP}\;\text{PPD}{\text{K}}_{\text{2}}\right]=0 \end{array}$$

Here p1 denotes the probability for a PPDK_2_ subunit near a bound PPDK_1_ subunit, and p2 is the same for a PPDK_1_ subunit near a bound PPDK_2_ subunit. The simplest model assumes that p1 and p2 are the fractions of the corresponding monomers

(16) $$\text{p1}=\text{PPD}{\text{K}}_{\text{2}}∕\text{PPD}{\text{K}}_{T}$$

(17) $$\text{p2}=\text{PPD}{\text{K}}_{\text{1}}∕\text{PPD}{\text{K}}_{T}$$

A more detailed model that takes into account the spatial configurations of PPDK_0_, PPDK_1_ and PPDK_2_ in the tetramer is provided in the next section. The detailed model shows similar results for robustness, and makes further predictions on the correlations of the states of adjacent monomers.

These equations were solved analytically using the fact that PPDK concentration is much higher than RP levels (more than a 100-fold higher [13,33]). The avidity effect allows us to assume that the on-rate for RP bound to one monomer to bind an adjacent monomer on the same tetramer is very large, due to the increased local concentration (kon2, kon3 >> kon1 PPDK*T*, kon4 PPDK*T*). Also, off-rates are assumed to be much faster than enzymatic reactions rates as is the case for most enzymes (in-vivo experiments suggest that full activation/de-activation occur on the scale of 10-60 minutes, therefore phosphorylation/de-phosphorylation rates are in the range of 1-10 [1/sec], compared to koff rates on the scale of 1 msec [13,20,11] thus V_p_, V_k _<< koff1, koff4). Analytical solution of the model was obtained using Mathematica 7.0.

A lower bound for the ratio of ternary to binary complexes is obtained by solving Eq.(15) at steady-state. We denote B1 as the binary complex [PPDK_1_ RP], B2 as the binary complex [PPDK_2_ RP] and T as the ternary complex [PPDK_1_ RP PPDK_2_]. Also, for simplicity we assume symmetrical rates on both branches (kon2 = kon3, koff2 = koff3)

(18) $$\left(\text{2}\;\text{koff2}+{\text{V}}_{\text{k}}+{\text{V}}_{\text{p}}\right)\text{T}=\text{kon2}\;\text{(p1 B1}+\text{p2 B2)}$$

Therefore, the fraction of ternary to binary complexes is bounded from below by

(19) $$\text{T}/\left(\text{B1}+\text{B2}\right)\geq \text{kon2}/(\text{2 koff2}+{\text{V}}_{\text{k}}+{\text{V}}_{\text{p}}\})\text{ min(p1},\text{p2)}$$

Since kon2 is very large compared to koff2 due to the avidity effect [22], kon2/koff2 = A >> 1, and one can neglect V_k_,V_p _compared to koff2 [13,11]

(20) T∕(B1+B2)≈Amin(p1,p2)>>1

This prevalence of the ternary complex breaks down only when the probabilities p1 or p2 become small, on the order of 1/A where A is estimated to be on the order of 100 [11,22,34].

### Solution of the product inhibition equation

To describe the output formation rate F as a function of the input (ADP levels) we used Eq. (5):

(21) $${\text{V}}_{\text{k}}\left(\text{ADP}\right)\left(\text{B1}+\text{T}\right)-{\text{V}}_{\text{p}}\left(\text{ADP}\right)\;\left(\text{B2}+\text{T}\right)=0$$

where V_k _and V_p _are effective phosphorylation and de-phosphorylation rates, B1 = [ PPDK_1_ RP] and B2 = [ PPDK_2_ RP] are the concentrations of the binary complexes and T = [ PPDK_1_ RP PPDK_2_] is the ternary complex concentration. Since V_k _is activated by ADP and V_p _is inhibited by ADP in a Michaelis-Menten fashion [17,35], we take the dependence on ADP levels to be

(22) $${\text{V}}_{\text{k}}∕{\text{V}}_{\text{p}}={\text{V}}_{\text{k}0}∕{\text{V}}_{\text{p}0}\text{ADP}$$

where V_k0 _and V_p0 _are effective rate constants dependent on enzyme catalytic rate and on/off rates.

Product-inhibition of PPi is formulated most generally as [19]:

(23) $$\text{Vp}=\left({\text{V}}_{\text{p}0}-{V^{\prime }}_{p}\text{PPi}∕{\text{K}}_{\text{i2},\text{PPi}}\right)∕\left(\text{1}+\text{PPi}∕{\text{K}}_{\text{i},\text{PPi}}\right)$$

where the catalytic rate and the binding are both lowered due to the presence of the reaction product. For clarity, we assume that the product inhibition is of a competitive type [17], where the product occupies the catalytic site thus preventing catalysis. The equations for the rates then simplify to

(24) $${\text{V}}_{\text{p}}={\text{V}}_{\text{p}0}∕\left(\text{1}+\text{PPi}∕{\text{K}}_{\text{i},\text{PPi}}\right)$$

For Figure 4, these equations were solved numerically for different concentrations of PPDK total amount using Mathematica 7.0.

We finally note that perfect robustness (complete insensitivity) to all protein and metabolites is an idealized feature. One may ask whether it persists if one adds additional reactions which have been neglected due to their small relative rates. We find (see additional file 2), that adding such reactions (e.g. the contribution of the binary complexes, the contribution of other reactions that make AMP and PPi) preserves approximate robustness: if the rates of these reactions are on order of a small number ε relative to the corresponding reactions above, sensitivity to proteins and metabolites is no longer strictly zero but is small, on the order of ε. We also note that the dependence of V_p_0 on Pi levels is neglected in this discussion due to the high and buffered Pi levels in the cell, making this metabolite unlikely to fluctuate as much as other metabolites [36,37].

### Spatial model of PPDK configurations

We developed a model in order to study the binding of RP to the four subunits of PPDK. For a ternary complex to form, RP must bind a PPDK_1_ and a PPDK_2_ that are neighboring subunits. It then can catalyze either phosphorylation or de-phosphorylation. Each PPDK subunit has three conformations possible: PPDK_0_, PPDK_1_ and PPDK_2_. Taking into account the symmetries of the tetramer there are 21 possible configurations.

The relative occupancy of the different configurations was calculated using a Master equation model, with transitions between configurations carried out by the binding of the enzyme RP, to a single domain or two adjacent domains. The single-domain binding events are essential to prevent the system from becoming stuck in an all PPDK_1_ or all PPDK_2_ state. The probability of state i is P(i), and the transition rate to state j is wij, and

(25) $$\frac{dP\left(i\right)}{dt}=\underset{j\neq i}{\sum }w_{ji}P\left(j\right)-\underset{j\neq i}{\sum }w_{ij}P\left(i\right)$$

The transition probabilities were calculated based on the number of adjacent PPDK_0_, PPDK_1_, PPDK_2_ and PPDK_1_ PPDK_2_ subunit pairs in the configuration. Each configuration has a probability μ to phosphorylate or de-phosphorylate any one of its subunits by single-subunit binding of RP, or RP can bind adjacent PPDK_1_ and PPDK_2_ subunits (if they exist in that configuration) and to either phosphorylate the PPDK_1_ subunit with a probability η1 or de-phosphorylate PPDK_2_ with a probability η2. Since the ternary complex is long-lived, the reaction almost always occurs before unbinding, and thus η1 and η2 approach unity. Also, in the binary complex, the probability for a reaction to occur before unbinding is the ratio of RP catalytic rate to the unbinding rate, so that:

(26) $$\mu /\eta_{\text{1},\text{2}}=\text{1}/{\text{2(V}}_{\text{k}}/(\text{koff1}+{\text{V}}_{\text{k}}{\text{) + V}}_{\text{p}}\text{/(koff4}+{\text{V}}_{\text{p}}))$$

The probability for an auto-phosphorylation reaction of a PPDK_0_ subunit is denoted by δ1 and the phospho-transfer reaction by δ2. Therefore, in order to have accumulation of PPDK_1_ and PPDK_2_ subunits, δ1 should be greater than δ2.

Solving the Master equation yields the fraction of each configuration at steady-state. We find that the avidity mechanism favors clustering of the PPDK_1_ and PPDK_2_ subunits. Thus, PPDK_1_ and PPDK_2_ subunits tend to be maximally spatially separated.

### Analytical results of the major configurations' occupation at steady-state

The following simplified model allows for an analytical estimate of the configurations distribution at steady-state for arbitrary protein's size. As shown from the solution of the Master equation (see previous section), the dominant configurations are ones where the modified and unmodified subunits cluster together and are phase separated. Therefore we consider only transitions between these states. The probability for a certain configuration state is denoted by its amount of modified subunits, namely, N(0) is the probability for the configuration where all subunits are unmodified, N(1) is the probability for a configuration with one modified subunit and so on. We further assume that for all states which are not fully modified or unmodified, reactions from the ternary complex are dominant and thus neglect binary reactions from these configurations. The modification reaction rate from a ternary complex is denoted by η1 and de-modification reaction rate from a ternary complex is denoted by η2. Similarly, modification and de-modification reaction rates from a binary complex are denoted by ε1 and ε2 respectively.

The model yields the following set of equations:

(27) dN(0)∕dt=-nε1 N(0)+2η2 N(1)=0

(28) dN(1)∕dt=nε1N(0)+2η2N(2)-2(η1+η2)N(1)=0

(29) $$\begin{array}{c} \text{d}\;\text{N(2)}∕\text{dt}=\text{2}\;\eta \text{2}\;\text{N(3)}+\text{2}\;\eta \text{1}\;\text{N(1)}-\text{2(}\eta \text{1}+\eta \text{2)}\;\text{N(2)}=0\\ \ldots \end{array}$$

(30) dN(n)∕dt=-nε2 N(n)+2η1N(n-1)=0

The general solution is easily admitted. When one assumes that ε1 = ε2 = ε and that η1 = η2 = η, the solution reduces to:

(31) N(0)=N(n)=η∕(2(n-1)ε+2η)

(32) N(i)=ε∕((n-1)ε+η),i=1,2…n-1

It is thus evident that the ratio between the boundary states to the 'bulk' (i.e. all configurations with partial number of modified subunits) is of order ε/η. This ratio can be viewed as the energetic cost of shifting the boundary between the two domains of modified and unmodified subunits. Also, it suggests (as the Master equation solution indeed indicates) that the neglected states with two or more boundaries between domains are of order (ε/η)^2 and higher, depending on the number of domains.

### Ternary to Binary reactions ratio is inversely dependent on the number of protein's subunits

The avidity effect stems from the ability of the bi-functional enzyme to bind neighboring modified and unmodified subunits. The modification or de-modification reactions are thus limited to proteins that have mixed pairs of modified and unmodified subunits. Here we solve a toy model of the avidity process to assess the dependence of the ratio of ternary to binary reactions on the number of protein subunits.

Following the analysis of configuration states (see above section), we assume that most proteins are phase separated (where a sequence of modified subunits is followed by a sequence of unmodified subunits). Hence, the number of modified subunits is characteristic of the protein state. We also assume that transitions between states with mixed subunits (modified and unmodified) are committed from a ternary complex due to the enhanced local concentration caused by the avidity effect. Therefore, modification and de-modification reactions occur only from the "edge" states when the protein's configuration is either fully modified or unmodified.

We define the mean number of steps to reach one of the boundaries (all modified/all unmodified) as M(i), meaning M(1) is the mean number of steps to reach the boundary from the state of one modified subunit, M(2) is the mean number of steps to reach the boundary from a two modified subunits state and so on up to M(N-1). Each state has a probability p to commit modification and (1-p) to commit de-modification.

Without loss of generality, we can assume that a binary reaction occurred from the 'all unmodified' configuration. Then, M(1) will reflect the ratio between ternary to binary reactions, assuming that ternary reactions have a much more faster time scale than binary reactions (due to avidity, the unbinding of the ternary complex is orders of magnitude slower). To calculate M(1) we solve a regression model, where each state is derived from the state next to it. For example, when the current state is one modified subunit, the mean number of reactions to reach one of the boundaries, M(1), is given by a probability (1-p) to reach the boundary by a de-modification (yielding the 'all unmodified' state) while with probability p the mean number of steps will be one plus the mean number of reactions from the state with 2 modified subunits. The equations to solve are thus:

(33) M(1)=(1-p)+p(1+M(2))

(34) $$\begin{array}{c} \text{M(2)}=\left(\text{1}-\text{p}\right)\left(\text{1}+\text{M(1)}\right)+\text{p(1}+\text{M(3))}\\ \ldots \end{array}$$

(35) M(N-1)=(1-p)(1+M(N-2))+p

This model can be solved analytically. For concreteness, we take p = 1/2 (a state where modification and de-modification are equally probable, i.e. near the system's steady-state). Then, M(1) simplifies to

(36) M(1)=N-1

Therefore, near the steady-state the ratio between ternary to binary reactions is proportional to the protein's number of subunits. To confirm this result, we run Monte-Carlo simulations where the number of proteins is two orders of magnitude larger than the number of bi-functional enzymes. We further assumed that there is an equal probability for modification and de-modification and that only states of fully modified and fully unmodified can react from a binary complex. The numerical results indeed show that the ratio between ternary and binary reactions is proportional to the number of protein's subunits and goes as (N-1) where N is the protein's subunits number.

## Competing interests

The authors declare that they have no competing interests.

## Authors' contributions

YH, AM, RM and UA conceived the study and participated in its design. YH, AM, RM and UA analyzed theoretical models and numerical simulations results. YH, AM, RM and UA drafted the manuscript. All authors have read and approved the final manuscript.

## Supplementary Material

Additional file 1 **Model equations for the PPDK system**. In this file we elaborate on the PPDK model's equations and their derivation. Click here for file

Additional file 2 **Robust Control in the Carbon Fixation Pathway of C**_**4 **_**Plants - Supplementary Information**. In this file we present further analysis of the model's stability and the effects of perturbations on robustness. We elaborate on the estimation of PEP formation rate and PPi sources in the chloroplast. We show the dependency of the ratio between ternary to binary complexes on avidity and modification rate and finally discuss a possible implication of the model to the metabolic pathway in bacteria. Click here for file
