# Supplementary material for: Robust Control of PEP Formation Rate in the Carbon Fixation Pathway of C4 Plants by a Bi-functional Enzyme
Source: BMC Syst Biol. 2011 Oct 24;5:171. doi: 10.1186/1752-0509-5-171 (PMC3240839; doi:10.1186/1752-0509-5-171)
Supplement: Additional file 1 — Model equations for the PPDK system. In this file we elaborate on the PPDK model's equations and their derivation. [file 1752-0509-5-171-S1.PDF]

**Additional file 1: Model equations for the PPDK system**

Here, we present the model of the PPDK system with its dynamic equations.

PPDK has three forms: $PPDK_0$, $PPDK_1$ and $PPDK_2$. Only $PPDK_1$ is active in catalyzing the production of PEP. $PPDK_1$ is formed by auto-phosphorylation of $PPDK_0$ (with ATP and Pi as substrates) at a rate $\beta_1$(Pi,ATP) and by de-phosphorylation of $PPDK_2$ by RP (with Pi as a substrate and ADP and PPi as effectors of the catalytic rate) at a rate: $V_p$(Pi,ADP,PPi).

$PPDK_1$ is converted back to $PPDK_0$ by phsophotransfer to pyruvate to form PEP at a rate $\beta_2$(pyruvate) , and by the phosphorylation of $PPDK_1$ by RP (which is dependent on ADP and Pi) at a rate $V_k$(ADP,Pi). RP can bind PPDK in either a binary complex (RP with one PPDK subunit) or a ternary complex (RP with two subunits on the same tetramer). Therefore, the rate of change of $PPDK_1$ concentration is

(1) $$d\,PPDK_1/dt = \beta_1(Pi,ATP)\,PPDK_0 + V_p(Pi,ADP,PPi)([PPDK_1\,RP\,PPDK_2] + [RP\,PPDK_2]) - $$

$$-\beta_2(pyruvate)\,PPDK_1 - V_k(ADP,Pi)([PPDK_1\,RP\,PPDK_2] + [RP\,PPDK_1])$$

The same considerations yield dynamic equations for $PPDK_0$ and $PPDK_2$

(2) $$d\,PPDK_0/dt = \beta_2(pyruvate)\,PPDK_1 - \beta_1(Pi,ATP)\,PPDK_0$$

(3) $$d\,PPDK_2/dt = V_k(ADP,Pi)\,([PPDK_1\,RP\,PPDK_2] + [RP\,PPDK_1]) - $$

$$-V_p(Pi,ADP,PPi)([PPDK_1\,RP\,PPDK_2] + [RP\,PPDK_2])$$

There are two conservation laws, for total PPDK and RP protein levels:

(4) $$PPDK_T = PPDK_0 + PPDK_1 + PPDK_2 + [PPDK_1\,RP] + [PPDK_2\,RP] + [PPDK_1\,RP\,PPDK_2]$$

(5) $$RP_T = [PPDK_1\,RP] + [PPDK_2\,RP] + [PPDK_1\,RP\,PPDK_2] + RP$$

The feedback loop in the model involves PPi which is formed by $PPDK_0$ auto-phosphorylation at a rate $\beta_1$(Pi,ATP) and by dephosphorylation of $PPDK_2$ at a rate $V_p$(Pi,ADP,PPi). PPi is also formed by other processes in the chloroplast at an effective rate denoted $\beta_{chl}$. PPi is removed at a rate $\alpha$, so that its dynamic equation reads

(6) $d\,[PPi]/dt = \beta_{chl} + \beta_1(Pi,ATP)\ PPDK_0 + V_p(Pi,ADP,PPi)\ [PPDK_1\ RP\ PPDK_2] - \alpha\ [PPi]$

As mentioned in the text, we neglect $\beta_{chl}$ assuming that the unusually high physiological concentration of PPDK makes it the most dominant producer of PPi (see Additional file 2 for more details). In addition, we neglect PPi production from the dephosphorylation of $PPDK_2$ because RP is more than 100 times less abundant than PPDK.

Other metabolites, such as Pi and ADP are assumed not to be significantly affected by their reactions with the enzyme RP (due to its small amounts and slow catalytic rate). Thus, they are held as parameters in the model.

Finally, the model requires equations for the formation of the binary and ternary complexes which have the following equations (see Fig 2b and the Methods section for more details),

(7) $d[PPDK_1\ RP]/dt = kon1\ PPDK_1\ RP - (koff1+Vk+kon2\ p1)\ [PPDK_1\ RP]+$
$+koff2[PPDK_1\ RP\ PPDK_2]$

(8) $d[PPDK_2\ RP]/dt = kon4\ PPDK_2\ RP - (koff4+Vp+kon3\ p2)\ [PPDK_2\ RP]+$
$+koff3[PPDK_1\ RP\ PPDK_2]$

(9) $d[PPDK_1\ RP\ PPDK_2]/dt= kon2\ p1\ [PPDK_1\ RP] +kon3\ p2\ [PPDK_2\ RP]-$
$-(koff2+ koff3+Vk+Vp)[PPDK_1\ RP\ PPDK_2]$

where p1 is the probability for a $PPDK_2$ site near a $PPDK_1$ site, and p2 is the same for a $PPDK_1$ site near a $PPDK_2$ site. To calculate p1 and p2 we used two approaches. In a mean field approach, $p1= PPDK_2/PPDK_T$ and $p2 = PPDK_1/ PPDK_{T.}$

We also calculated p1 and p2 from a detailed model for the configurations on a PPDK tetramer. Both approaches yield qualitatively similar results. Avidity is important in the present context, since it makes kon2 and kon3 at least 100-fold larger than kon1 and kon4.

We solve the model at steady-state, where all dynamic equations equal zero to find the steady-state solution for the output of the system: the PEP production rate by $PPDK_1$.
