# Supplementary material for: Robust Control of PEP Formation Rate in the Carbon Fixation Pathway of C4 Plants by a Bi-functional Enzyme
Source: BMC Syst Biol. 2011 Oct 24;5:171. doi: 10.1186/1752-0509-5-171 (PMC3240839; doi:10.1186/1752-0509-5-171)
Supplement: Additional file 2 — Robust Control in the Carbon Fixation Pathway of C4 Plants - Supplementary Information. In this file we present further analysis of the model's stability and the effects of perturbations on robustness. We elaborate on the estimation of PEP formation rate and PPi sources in the chloroplast. We show the dependency of the ratio between ternary to binary complexes on avidity and modification rate and finally discuss a possible implication of the model to the metabolic pathway in bacteria. [file 1752-0509-5-171-S2.PDF]

Robust Control of PEP Formation Rate in the Carbon Fixation Pathway of C_4 Plants by a Bi-functional Enzyme - Supplementary Information

Yuval Hart[1], Avi Mayo[1], Ron Milo[2] and Uri Alon[1]

[1]Dept. of Molecular Cell Biology, [2] Dept. of Plant Sciences,

Weizmann Institute of Science, Rehovot Israel 76100

Table of Contents

Stability analysis of the model's solutions 1
Estimation of the PEP formation flux 3
Sources of PPi in the chloroplast 3
ADP and other adenylates regulators effect on the robust solution 4
Perturbations effect on the robust solution 4
Ternary vs. Binary complexes maximal ratio depends linearly on the avidity strength and on the modification reaction rate 5
Measurements of Ohta et al. indicate low variability in PPDK content before transformation and high variability after transformation 7
PPDK involvement in bacteria's glycolysis pathway suggests a mechanism for a robust flux of ATP formation 7

Stability analysis of the model's solutions

The model's general equations for the rates of phosphorylation and de-phosphorylation with the competitive inhibition effect of their products [1] are as follows:

(1) $Vk = Vk0 / (1+AMP/Ki_{,AMP})$

(2) $Vp = Vp0 / (1+PPi/Ki_{,PPi})$

where $Ki_{,PPi}$ and $Ki_{,AMP}$ are the inhibition constants of the PPi and AMP on the rates of dephosphorylation and phosphorylation, Vp0 and Vk0 respectively. For brevity we denote for later use $\gamma = \frac{K_{i,PPi}}{K_{i,AMP}}$.

The mathematical model presented above has three fixed points:
F=0, F α $PPDK_T$ and $F=F^*= (V_{ko}- V_{po})/(v_{po} / (\alpha1\ Ki_{,AMP}) – V_{ko}/ (\alpha2\ Ki_{,PPi}))$. A stability check for the three solutions was made by a linearization of the dynamic equation for the change in $PPDK_2$. The analysis indicates that there are three regimes of stability, each one with only one stable fixed point (subject to the demand that total PPDK amounts are conserved):

(3) F=0 ⇔ Vk0>Vp0

(4) $F \alpha PPDK_T \Leftrightarrow \gamma<1$ & $Vk0<\gamma(\alpha1\ Ki_{,AMP}+PPDK_T)/(\alpha2\ Ki_{,PPi}+PPDK_T)Vp0$

or

$\gamma>1$ & $Vk0<Vp0$

(5) $F=F^* \Leftrightarrow \gamma<1$ & $\gamma(\alpha1\ Ki_{,AMP}+PPDK_T)/(\alpha2\ Ki_{,PPi}+PPDK_T)Vp0<Vk0<Vp0$

The phase diagram of the generalized system is presented below, for two different levels of total PPDK amount. For moderate levels of ADP (Region II, see text for conditions for the break of robustness) the flux is robust and the two curves of PEP formation flux coincide. For high ADP levels (darkness, region III) both of the systems are shut and a low basal level of flux is possible. At very low ADP levels (high light conditions, region I) the flux is at its maximum and all PPDK is activated yielding dependence between PEP formation flux and total PPDK amounts as shown on Figure S1.

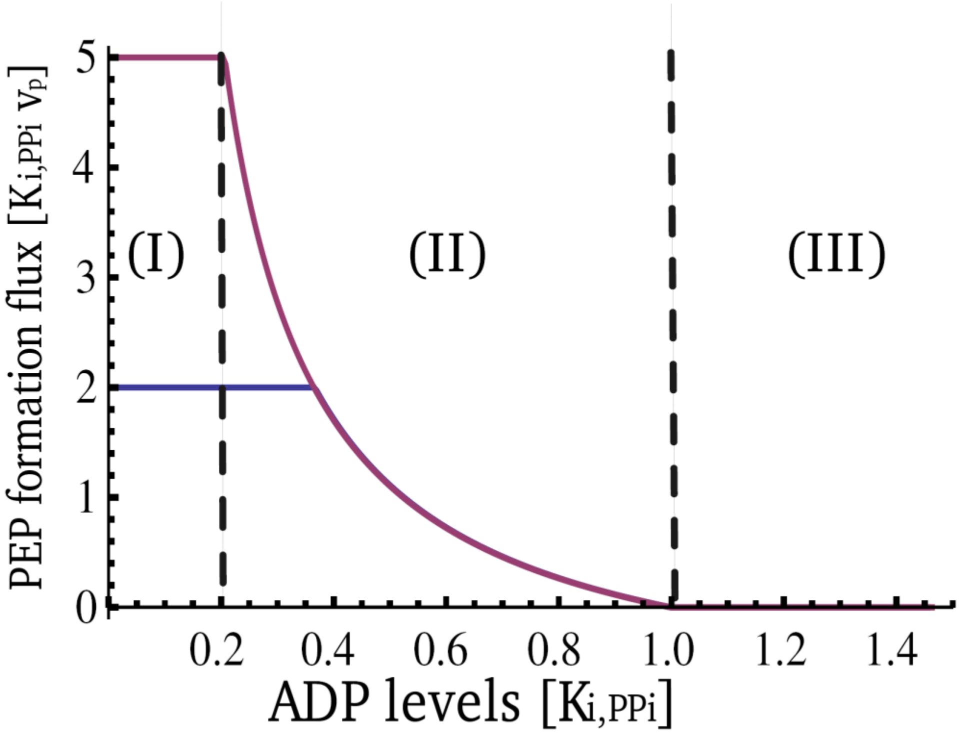

Figure S1: **The PEP formation flux is robust to PPDK levels and sensitive to ADP levels**. The robust solution has a range of validity. At region I, ADP levels are very low and $Vk0< \gamma(\alpha1\ Ki_{,AMP}+PPDK_T)/(\alpha2\ Ki_{,PPi}+PPDK_T)Vp0$. In this case, the flux is fully dependent on PPDK levels. Once ADP levels are high enough (region II) such that Vk0 is bigger than the lower bound (see text), the robust solution holds and it is a function of Vk0 and Vp0 and so it depends on ADP levels. When ADP levels are so high that Vk0>Vp0 then all PPDK subunits are inactive and the PEP formation flux is set to a very low basal level (region III). Two curves of different total PPDK levels are plotted, where the ratio between blue curve and purple curve is set to 2.5. The entry to the robust regime is dependent on PPDK levels also but only weakly once $PPDK_T >> Ki_{,AMP}, Ki_{,PPi}$. Other model parameters are as described in Fig.2 in the text.

Estimation of the PEP formation flux

The avidity model predicts a flux that is dependent on two biochemical parameters: Ki,PPi, the inhibition constant of PPi on RP and α, the degradation rate of PPi. Measurements of the two indicate that Ki,PPi = 160μM [1] and phosphatases specific activity is about 40 μmol (Pi)/mg chl/min [2].
We start by converting the phosphatases specific activity to the rate α

(6) $Specific\ activity = 40 \frac{Pi\ \mu mol}{mg\ chl*min}$

Chlorophyll molecular weight is 868 thus, in 1 mg of chlorophyll there are ~1 μmol of chlorophyll molecules. The chloroplast volume is about, $V_{cho} \sim 20\ \mu m^3$ [3], while chlorophyll concentration in the chloroplast is 30 mM [4]. This yields 20*30*10^6=6*10^8 molecules in a chloroplast. Therefore, there is $R = \frac{6\ 10^8 [chlorophyll\ molecules\ per\ chloroplast\ ]}{6\ 10^{17}\ [chlorophyll\ molecules\ per\ mg\ chlorophyll]} = 10^{-9}$ mg chlorophyll per chloroplast.
Thus, at the chloroplast, saturating amounts of substrate (PPi) results in concentration buildup rate of product (Pi) that equals

(7)

$$k_{cat}\,[E_T] = \frac{Specific\ \ Activity\ *R}{V_{cho}} = 20 \frac{\mu mol\ *R}{mg\ chl*min\ *V_{cho}} = \frac{20\ 6\ 10^{17} molecules\ *10^{-9}\ \frac{mg\ chl}{cho}}{60\ sec*\ mg\ chl*20\ \mu m^3} =$$
$$= \ 10^7\ \frac{molecules}{sec\ *\ \mu m^3}$$

To calculate the rate α, which is assumed to have first-order kinetics, i.e. [PPi]<<Km, (and so $k_{cat}\,[E_T]\frac{s}{s+K_m} \sim \frac{k_{cat}\,[E_T]}{K_m} s = \alpha\ s$) where Km of phosphatases is taken to be on the order of 100 μM (Km of pyrophosphatases of sugar cane is 0.75mM [5] and in sorghum is 0.035mM [6]),

(8) $\alpha = \frac{k_{cat}[E_T]}{K_m} = \frac{10^7 \frac{molecules}{sec\ *\mu m^3}}{0.1\ mM} = \frac{10^7}{0.1\ 10^6} = 100 \frac{1}{sec}$

Using this rate to calculate the order of magnitude of PEP formation rate yields

(9) $F_0 = \alpha\ K_{i,PPi} = 100 \frac{1}{sec} 160 \mu M$

$$\rightarrow \frac{\#Reactions}{sec\ chloroplast} = F_0 V_{cho} {\sim} 100 \frac{1}{sec} * 20\ \mu m^3\ * 160 \mu M * 10^3 \frac{molecules}{\mu M\ \mu m^3}$$
$$\sim\ 10^8 \frac{molecules}{\sec chloroplast}$$

Thus, PEP formation flux is of the order of 10^8 reactions per second per chloroplast. This estimate agrees well with estimation of the photosynthetic flux, where a rate of 200-500 μmol (CO_2) per hour per mg chlorophyll is measured [7, 8], yielding an estimation of ~$10^7 - 10^8$ carbons assimilated per second per chloroplast.

Sources of PPi in the chloroplast

In order for the model to be physiologically relevant, PPi levels, being the major signal carrier of the PEP formation flux and PPDK activity, have to reflect accurately the flux of the auto-kinase reaction of PPDK. We note that while two

isoforms of PPDK are known to exist (cytosolic and plastidic isoforms [9-11]), we focus on the plastidic isoform which is relevant to C_4 metabolism.

As shown, the order of magnitude of this flux which is eventually responsible for the carbon assimilation process is ~ 10^8 reactions per second per chloroplast. Since this flux is coupled to the assimilation of carbon atoms, other processes that are involved in protein synthesis or proteins degradation are estimated to be on the order of 1000 times or more less frequent when estimating the average carbon content of each protein.

An important competing process in the cell is the charging of tRNA in the translation process. Contrary to proteins which have a large carbon content, amino-acids have about 5 carbons per amino-acid and the translation process is vastly used in the cell. During most of these processes an ATP and Pi molecules are used to charge the tRNA and produce AMP and PPi, making these processes an amenable candidate for a PPi source.

To check if indeed this is the case, one should remember that the chloroplast is a separated organelle and that its envelope is not permeable to PPi molecules. Since most of translation is conducted outside the chloroplast (only few genes are transcribed and translated in the chloroplast itself), the source's rate is dependent on the transport rate of PPi across the chloroplast envelope. This rate was measured to be in the order of ~1-2 μmole/mg chl/ per hour [12] and thus is almost two orders of magnitude lower than the production rate of PPi from the PPDK pathway. It is thus reasonable to conclude that most of PPi in the chloroplast originates from the carbon assimilation pathway and not other processes.

ADP and other adenylates regulators effect on the robust solution

While ADP is considered to be a dominant regulator of PPDK activity, both other adenylates (AMP and ATP) have been shown to have some effect on RP phosphorylation/dephosphorylation rates (see [9, 13] and references therein). These effects however change only the encoded PEP formation rate value, as dictated by the rates to be:

(10) $F^* = \alpha\, K_{i,PPi}\, (V_{p0}(ADP,ATP,AMP)/V_k(ADP,ATP,AMP)-1)$

and not the robustness of the formation rate. See also next section which deals with adding perturbations to the rates or adding other processes affecting regulators levels.

Perturbations effect on the robust solution

We note that the perfect robustness (complete insensitivity) to all protein and metabolites is an idealized feature. One may ask whether it persists if one adds additional reactions which have been neglected due to their small relative rates. We find that adding such reactions (e.g. the contribution of the binary complexes, the contribution of other reactions that produce AMP and PPi and other product inhibition reactions) preserves approximate robustness: if the rates of these reactions are of order of a small number ε relative to the corresponding reactions above, sensitivity to proteins and metabolites is no longer strictly zero but is rather small, on the order of ε.

We also calculated the sensitivity of PEP formation flux to total PPDK levels, $d\, Log(PPDK_1)/d\, Log(PPDK_T)$. This calculation was done to both the product inhibition solution without accounting for an avidity effect (only binary reactions are allowed) and for a model having both ternary and binary reactions, where the ratio of

binary to ternary reactions is ε. For concreteness, we calculated the sensitivity of all models at $PPDK_T$ levels where the robust solution is attained (the crossing point of all graphs depicted at figure S2).One finds that the sensitivity of the product inhibition model vs. the perturbed avidity model is linear in ε at small ε values and is given by:

$$(11)\ S_{avidity}/S_{PI} = 2(3v1-1)\,\varepsilon / (-1+v1-2\,\varepsilon+6\,v1\,\varepsilon)$$

where v1 is the ratio of the effective maximal de-modification to modification rates. At the extreme case where all configurations are at a bimodal distribution and ε levels are of order 0.3, this ratio yields a sensitivity reduction by a factor of 0.643.

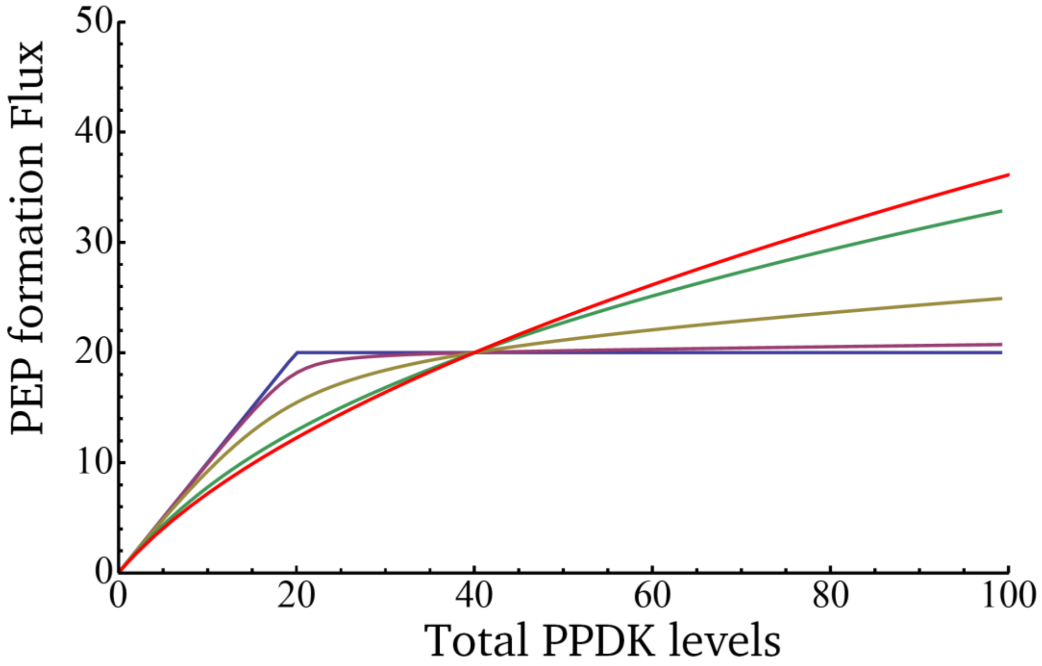

Figure S2: **The robust solution of the avidity model scales linearly with the strength of the perturbations.** The solution of the avidity model is presented with an addition of the binary reactions. Blue line – PEP formation flux at different total PPDK levels, the avidity model was calculated without reactions from the binary complexes. Red line - PEP formation flux at different total PPDK levels, only binary reactions were considered, the solution reflects the product inhibition of the flux on the phosphatase activity. Purple, Yellow and Green lines – PEP formation flux at different total PPDK levels, reactions are considered both from the ternary and binary complexes where the relative strength of the binary reaction was taken to be: 0.01,0.1,1 respectively.

Ternary vs. Binary complexes maximal ratio depends linearly on the avidity strength and on the modification reaction rate

We analyzed numerically the maximal ratio between the ternary and binary complexes, using the following mass-action kinetics scheme (see also the Methods section in the MS):

(12) $d[PPDK_1\ RP]/dt = kon1\ PPDK_1\ RP - (koff1+Vk+kon2\ p1)\ [PPDK_1\ RP] +koff2[PPDK_1\ RP\ PPDK_2] = 0$

(13) $d[PPDK_2\ RP]/dt = kon4\ PPDK_2\ RP - (koff4+Vp+kon3\ p2)\ [PPDK_2\ RP] +koff3[PPDK_1\ RP\ PPDK_2] = 0$

(14) $d[PPDK_1\ RP\ PPDK_2]/dt= kon2\ p1\ [PPDK_1\ RP] +kon3\ p2\ [PPDK_2\ RP]- (koff2+ koff3+Vk+Vp)[PPDK_1\ RP\ PPDK_2]=0$

Here p1 denotes the probability for a $PPDK_2$ subunit near a bound $PPDK_1$ subunit, and p2 is the same for a $PPDK_1$ subunit near a bound $PPDK_2$ subunit. The simplest model assumes that p1 and p2 are just the fractions of the corresponding monomers

(15) $p1 = PPDK_2/PPDK_T$

(16) $p2 = PPDK_1/ PPDK_T$

The ratio between ternary and binary complexes is highest at the point where p1=p2=1/2. We solved numerically for a wide range of parameters and got that the maximal ratio between ternary and binary complexes depends linearly on both the avidity strength (kon2/koff2) and on the ε, the probability for a reaction to occur from the binary complex. ε can be approximated by the modification reaction rate divided by the unbinding rate (ε ≈ Vk/koff1+Vp/koff4).

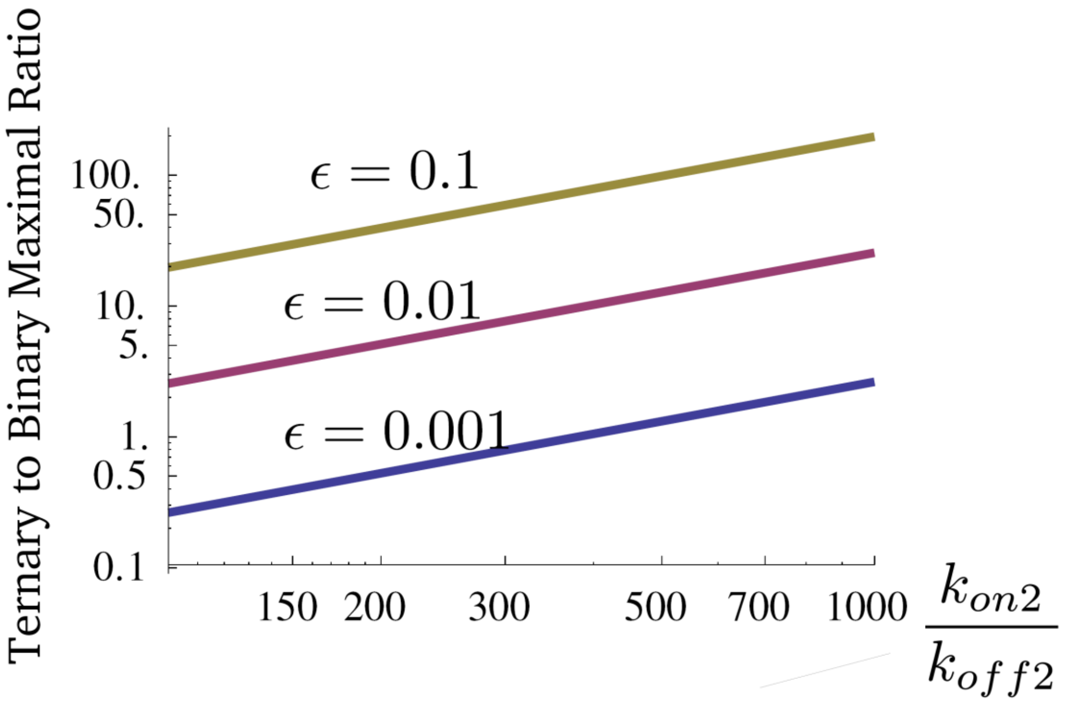

Figure S3: **Avidity and single binding reaction events probability control the dominance of the ternary complex**. The maximal ratio between the ternary complex and the binary ones is plotted as a function of the avidity enhancement factor, kon2/koff2. Numerical analysis shows that the maximal ratio between ternary and

binary complexes is proportional to ε kon2/koff2, where ε is the probability for a reaction to occur from a binary complex and can be approximated by ε ≈ Vk/koff1+Vp/koff4.

Measurements of Ohta et al. indicate low variability in PPDK content before transformation and high variability after transformation

Measurements of PPDK levels variation among different transformants at same growth conditions show a CV of ~10% [14]. It is thus compelling to assume that the significance of the regulatory system is aimed at resolving fluctuations in substrate levels (ATP, pyruvate) as well as responding to a change in protein levels that occurred due to a change of growth conditions (light-shadow effects).
It is interesting to note that the majority of successful transformations of the mutant gene in Ohta et al. experiments [14] exhibit expression levels that lay in the robust regime. It is only 1/12 of the transformants that exhibit a dependency on total amount of the enzyme PPDK. This may suggest that the robust mechanism is beneficial also from an evolutionary point of view accounting for the variability between individuals and not only that between cells and environmental conditions.

PPDK involvement in bacteria's glycolysis pathway suggests a mechanism for a robust flux of ATP formation

The PPDK enzyme and its regulation are thought to originate from bacteria ([15], [16] and references therein). There it is commonly used in the glycolysis pathway. Bacteria differ in several important features of the system: ATP instead of ADP is the second phosphorylation substrate, PPDK is a dimer and the phospho-transfer reaction is done at 3 sequential steps. These suggest that a different mechanism exists in bacteria where a possible robust mechanism may provide a balance between pyruvate production and PEP consumption according to ATP levels in the cell or control the flux of ATP formation similar to the mechanism suggested for the IDH/IDHKP system in E.coli [17]. The importance of PEP to cell's viability dictates a balance between the two. Growth rate measurements may indicate if indeed robustness is attained in primer organisms as well.

References

1. Burnell JN, Hatch MD: Regulation of C4 photosynthesis: purification and properties of the protein catalyzing ADP-mediated inactivation and Pi-mediated activation of pyruvate,Pi dikinase. *Arch. Biochem. Biophys* 1985, 237:490-503.

2. Kanai R, Edwards GE: The Biochemistry of C4 Photosynthesis. In *C4 Plant Biology*. San Diego: Academic Press; 1999:49-87.

3. Orth GM, Cornwell DG: The particle-volume distribution in purified corn-seedling chloroplasts. *Biochimica et Biophysica Acta* 1961, 54:389-391.

4. Nobel PS: *Physicochemical and Environmental Plant Physiology, Fourth Edition*. 4th edition. Academic Press; 2009.

5. Bucke C: The distribution and properties of alkaline inorganic pyrophosphatase from higher plants. *Phytochemistry* 1970, 9:1303-1309.

6. Krishnan VA, Gnanam A: Properties and regulation of Mg2+-dependent chloroplast inorganic pyrophosphatase from Sorghum vulgare leaves. *Archives of Biochemistry and Biophysics* 1988, 260:277-284.

7. Milo R, Jorgensen P, Moran U, Weber G, Springer M: BioNumbers--the database of key numbers in molecular and cell biology. *Nucleic Acids Res* 2010, 38:D750-753.

8. Usuda H: Adenine Nucleotide Levels, the Redox State of the NADP System, and Assimilatory Force in Nonaqueously Purified Mesophyll Chloroplasts from Maize Leaves under Different Light Intensities. *Plant Physiol* 1988, 88:1461-1468.

9. Chastain CJ, Chollet R: Regulation of pyruvate, orthophosphate dikinase by ADP-/Pi-dependent reversible phosphorylation in C3 and C4 plants. *Plant Physiology and Biochemistry* 2003, 41:523-532.

10. Ku MSB, Kano-Murakami Y, Matsuoka M: Evolution and Expression of C4 Photosynthesis Genes. *Plant Physiol.* 1996, 111:949-957.

11. Parsley K, Hibberd JM: The Arabidopsis PPDK gene is transcribed from two promoters to produce differentially expressed transcripts responsible for cytosolic and plastidic proteins. *Plant Mol Biol* 2006, 62:339-349.

12. Lunn JE, Douce R: Transport of inorganic pyrophosphate across the spinach chloroplast envelope. *Biochem. J* 1993, 290 (Pt 2):375-379.

13. Leegood RC, Walker RP: Regulation of the C4 Pathway. In *C4 Plant Biology*. San Diego: Academic Press; 1999:89-131.

14. Ohta S, Ishida Y, Usami S: High-Level Expression of Cold-Tolerant Pyruvate, Orthophosphate Dikinase from a Genomic Clone with Site-Directed Mutations in Transgenic Maize. *Molecular Breeding* 2006, 18:29-38.

15. South DJ, Reeves RE: Pyruvate, orthophosphate dikinase from Bacteroides symbiosus. *Meth. Enzymol* 1975, 42:187-181.

16. Burnell JN, Chastain CJ: Cloning and expression of maize-leaf pyruvate, Pi dikinase regulatory protein gene. *Biochemical and Biophysical Research Communications* 2006, 345:675-680.

17. Shinar G, Rabinowitz JD, Alon U: Robustness in Glyoxylate Bypass Regulation. *PLoS Comput Biol.* 2009, 5:e1000297.
